# Supplementary material for: Modeling the future of HIV in Turkey: Cost-effectiveness analysis of improving testing and diagnosis
Source: PLoS One. 2023 Jun 30;18(6):e0286254. doi: 10.1371/journal.pone.0286254 (PMC10313051; doi:10.1371/journal.pone.0286254)

**Supplemental Appendix**

In this appendix, we present additional data on surveillance values and model inputs. We also present the mathematical model formulation and model flow chart.

**HIV in Turkey:**


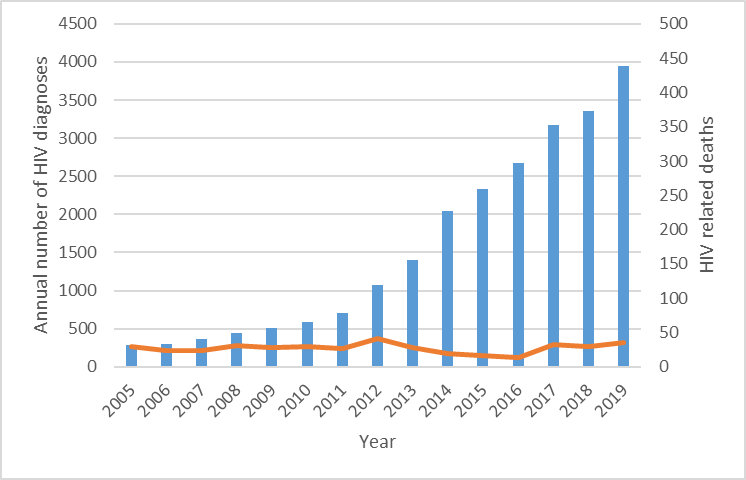


**S1 Fig.** The number of reported HIV diagnoses and HIV-related deaths in Turkey, 2005-2019 (Source: Turkish Ministry of Health. HIV-AIDS Statistics 2020).

**Model Summary:**


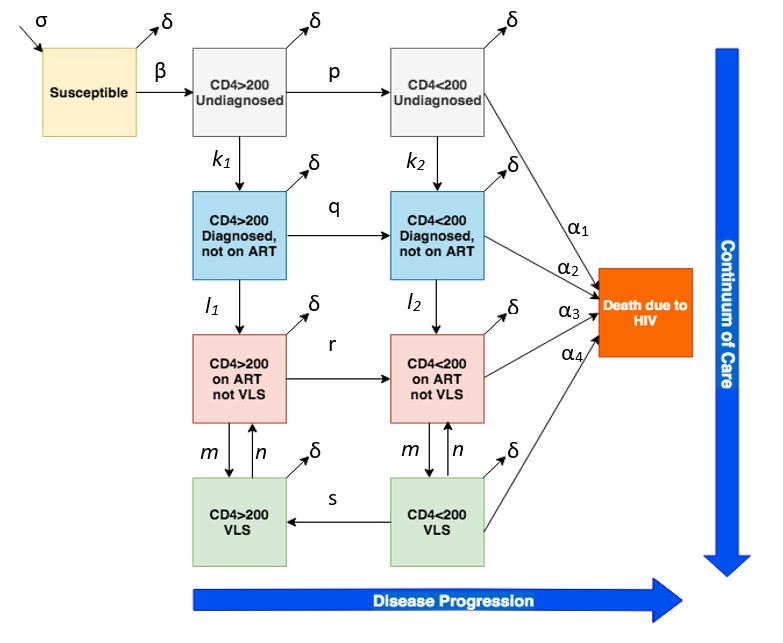


**S2 Fig.** Model flow diagram with notations.

**Data Summary:**

**S1 Table.** Key characteristics of patient cohorts.

| **Patient Cohort 1 (n=3242)** | **Value** | **Percentage (%)** |  | **Value** | **Percentage (%)** |
| --- | --- | --- | --- | --- | --- |
| Gender |  |  | Jail inmate |  |  |
| Female | 368 | 11 | Yes | 24 | 0.7 |
| Male | 2874 | 89 | No | 1933 | 59 |
| Age group |  |  | Missing/Unknown | 1295 | 40 |
| 18-25 years | 190 | 6 | CD4 count |  |  |
| 26-40 years | 1616 | 50 | Median at diagnosis | 379 |  |
| 41-65 years | 1314 | 40 | Median at 24 weeks | 494 |  |
| >65 years | 132 | 4 | Median at 48 weeks | 533 |  |
| Education level |  |  | Plasma RNA viral load at diagnosis |  |  |
| No education | 819 | 25 | <400 copies/ml | 209 | 6 |
| Literacy | 26 | 1 | 400-1000 copies/ml | 40 | 1 |
| Primary school | 304 | 9 | 1001-10000 copies/ml | 222 | 7 |
| Secondary school | 355 | 11 | 10001-100000 copies/ml | 997 | 31 |
| High school | 372 | 11 | 100001-1 million copies/ml | 1066 | 33 |
| University | 805 | 25 | >1 million copies/ml | 410 | 13 |
| Higher education | 411 | 13 | Missing/Unknown | 308 | 9 |
| Missing/Unknown | 149 | 5 | Plasma RNA viral load at 24 weeks |  |  |
| Marital status |  |  | <400 copies/ml | 761 | 23 |
| Single | 1659 | 51 | 400-1000 copies/ml | 29 | 0.9 |
| Married | 996 | 31 | 1001-10000 copies/ml | 24 | 0.7 |
| Divorced or widowed | 199 | 6 | 10001-100000 copies/ml | 9 | 0.3 |
| Missing/Unknown | 398 | 12 | 100001-1 million copies/ml | 9 | 0.3 |
| Health Insurance |  |  | >1 million copies/ml | 2 | 0.1 |
| Governmental ins. | 2085 | 64 | Missing/Unknown | 2418 | 74 |
| Private insurance | 10 | 0 | On treatment |  |  |
| No insurance | 63 | 2 | Yes | 1336 | 41 |
| Other | 101 | 3 | No | 1916 | 59 |
| Missing/Unknown | 993 | 31 | Race/ethnicity |  |  |
| Transmission category |  |  | White | 3235 | 99 |
| MSM | 903 | 28 | Black | 16 | 0.5 |
| PWID | 11 | 0.3 |  |  |  |
| MSM & HET | 362 | 11 |  |  |  |
| HET | 1197 | 37 |  |  |  |
| Missing/Unknown | 790 | 24 |  |  |  |

| **Patient Cohort 2 (n=4758)** | **Value** | **Percentage (%)** |  |  |  |
| --- | --- | --- | --- | --- | --- |
| Gender |  |  | Plasma RNA viral load at diagnosis |  |  |
| Female | 598 | 13 | <400 copies/ml | 332 | 7 |
| Male | 4160 | 87 | 400-1000 copies/ml | 56 | 1 |
| Age group |  |  | 1001-10000 copies/ml | 337 | 7 |
| 18-25 years | 232 | 5 | 10001-100000 copies/ml | 1431 | 30 |
| 26-40 years | 2271 | 48 | 100001-1 million copies/ml | 1506 | 32 |
| 41-65 years | 1994 | 42 | >1 million copies/ml | 600 | 13 |
| >65 years | 248 | 5 | Missing/Unknown | 496 | 10 |
| Transmission category |  |  | Plasma RNA viral load at 24 weeks |  |  |
| MSM | 1572 | 33 | <400 copies/ml | 2905 | 61 |
| PWID | 0 | 0 | 400-1000 copies/ml | 85 | 2 |
| MSM & HET | 27 | 1 | 1001-10000 copies/ml | 123 | 3 |
| HET | 2495 | 52 | 10001-100000 copies/ml | 64 | 1 |
| Missing/Unknown | 664 | 14 | 100001-1 million copies/ml | 38 | 1 |
| CD4 count |  |  | >1 million copies/ml | 14 | 0 |
| Median at diagnosis | 372 |  | Missing/Unknown | 1529 | 32 |
| Median at 24 weeks | 514 |  | On treatment |  |  |
| Median at 48 weeks | 578 |  | Yes | 4460 | 94 |
|  |  |  | No | 298 | 6 |

| **Patient Cohort 3 (n=556)** | **Value** | **Percentage (%)** |  |  |  |
| --- | --- | --- | --- | --- | --- |
| Transmission category |  |  | CD4 count |  |  |
| MSM | 163 | 29 | <100 | 10 | 2 |
| PWID | 10 | 2 | 100 – 200 | 100 | 23 |
| HET | 287 | 52 | 200 – 300 | 75 | 17 |
| Missing/Unknown | 96 | 17 | >300 | 255 | 58 |
| On treatment |  |  |  |  |  |
| Yes | 450 | 81 |  |  |  |
| No | 106 | 19 |  |  |  |

**Costs:**

The two main sources of cost calculations were the relevant studies from the literature and the datasets from the two main cohorts in Turkey-HIV-IST and HIV-TR. ART costs were defined based on the current drug costs and treatment regimens administered. Treatment regimens taken from the datasets were categorized and ranked by their frequencies. Then, the most common treatment regimens chosen were considered for calculating ART costs. After estimating annual ART costs, direct and indirect costs, our focus was to find HIV costs per patient considering both their continuum of care and CD4 level. Based on the continuum of care level, the unaware compartment includes only the non-HIV medication costs for comorbidities while the aware, not on ART compartments include the inpatient HIV cost, outpatient HIV cost, non-HIV medication cost for comorbidities, and lab costs. Two compartments (on ART, not VLS and VLS) include only ART costs.

These costs were obtained from the sources for 3 CD4 levels which were as follows: less than 100, between 100 and 300, and lastly greater than 300 cells/mm^3^. Since our model consisted of 2 CD4 levels (less than 200 and greater than 200), we needed to allocate the costs into 2 groups. First of all, we extended the CD4 level categorization as less than 100, between 100 and 200, between 200 and 300, and greater than 300 cells/mm^3^. Then, we used the percentages of patients with baseline CD4 counts in these 4 categories from the HIV-IST and HIV-TR data sets; the relevant percentages were 10%, 11%, 15%, and 63%, respectively. In other words, 22% of diagnosed patients had baseline CD4 levels less than 200 cells/mm^3^and 78% had CD4 levels greater than 200 cells/mm^3^. Then, we calculated the overall rates for these two CD4 categories. Our results were as follows:

- Among patients with CD4 levels of less than 200 cells/mm^3^

49% had a CD4 level of less than 100 cells/mm^3^

51% had a CD4 level between 100 and 200 cells/mm^3^

- Among patients with CD4 levels greater than 200 cells/mm^3^

19% had a CD4 level between 200 and 300 cells/mm^3^

81% had a CD4 level greater than 300 cells/mm^3^

As a final step, we calculated the weighted average of costs among patients with a CD4 level of less than 200 cells/mm^3^ within the category. Then, we applied the same process for those patients with a CD4 level greater than 200 cells/mm^3^ within the category. In other words, we found the average of direct cost values multiplied by their rates for each compartment among the two CD4 levels in the model, separately. Indirect costs were calculated using the same steps including the productivity loss cost in the calculations.

**S2 Table.** HIV costs in Turkey are based on a 3% discount rate.


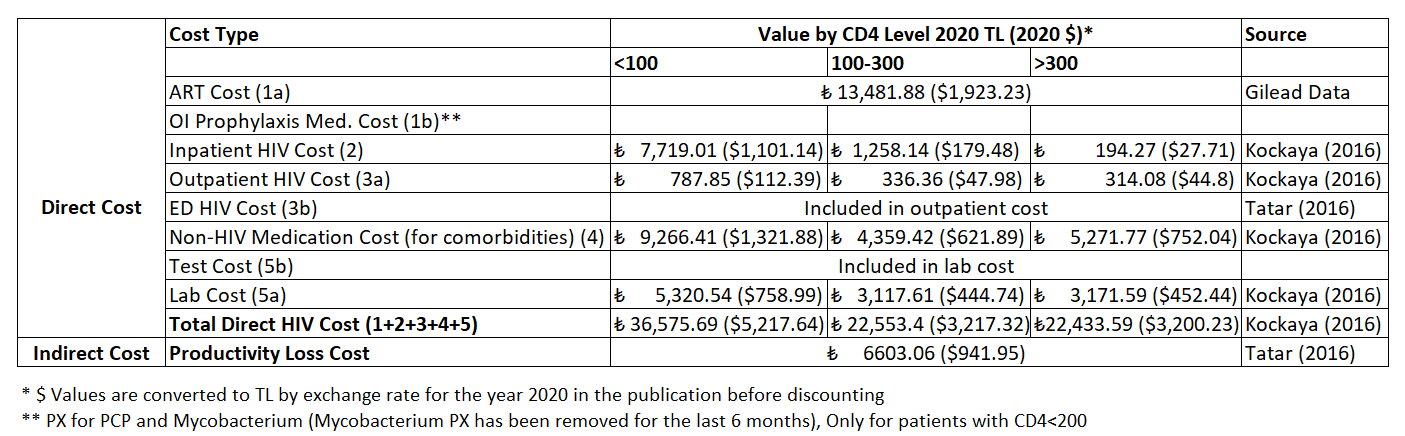


Based on the above table, we calculated the direct cost that would be associated with each compartment in the model (S3 and S4 Tables).

**S3 Table.** Direct cost in the model.


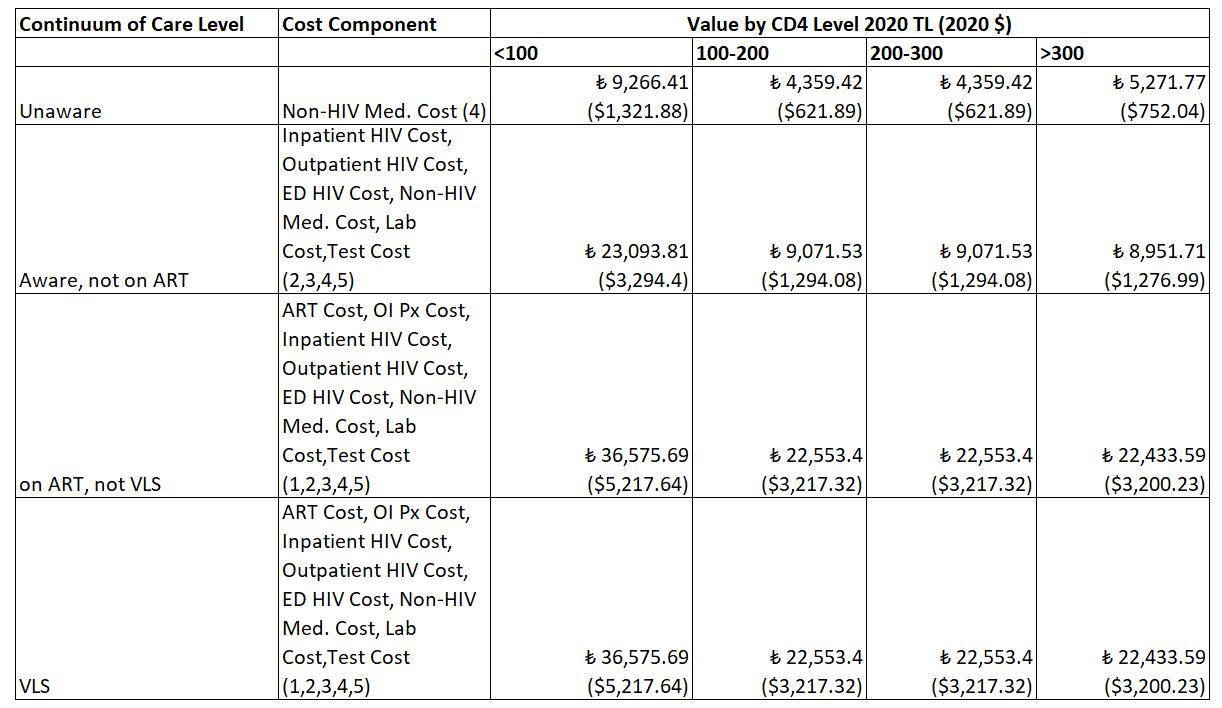


**S4 Table.** Direct cost by CD4 value applied in the model.

| **Continuum of Care** | **Value by CD4 Level 2020 TL (2020 $)** | |
| --- | --- | --- |
|  | **<200** | **>200** |
| **Unaware** | ₺ 6,760.6 ($964.42) | ₺ 5,098.67 ($727.34) |
| **Aware, not on ART** | ₺ 15,933.16 ($2,272.92) | ₺ 8,974.44 ($1,280.23) |
| **on ART, not VLS** | ₺ 29,415.04 ($4,196.15) | ₺ 22,456.32 ($3,203.47) |
| **VLS** | ₺ 29,415.04 ($4,196.15) | ₺ 22,456.32 ($3,203.47) |

**Dynamic compartmental model:**

The model formulation is summarized below:

$\frac{dS_{i}}{dt}=\sigma S_{i}-\frac{S_{i}\left[\beta_{1}UA_{i}+\beta_{2}DA_{i}+\beta_{3}TA_{i}+\beta_{1}US_{i}+\beta_{2}DS_{i}+\beta_{3}TS_{i}\right]}{N}-\delta S_{i}$ (1)

$\frac{dUA_{i}}{dt}=\frac{S_{i}[\beta_{1}UA_{i}+\beta_{2}DA_{i}+\beta_{3}TA_{i}+\beta_{1}US_{i}+\beta_{2}DS_{i}+\beta_{3}TS_{i}]}{N}-k_{1}UA_{i}-pUA_{i}-\delta UA_{i}$ (2)

$\frac{dDA_{i}}{dt}=k_{1}UA_{i}-l_{1}DA_{i}-qDA_{i}-\delta DA_{i}$ (3)

$\frac{dTA_{i}}{dt}=l_{1}DA_{i}+nVA_{i}-rTA_{i}-mTA_{i}-\delta TA_{i}$ (4)

$\frac{dVA_{i}}{dt}=mTA_{i}+sVS_{i}-nVA_{i}-\delta VA_{i}$ (5)

$\frac{dUS_{i}}{dt}=pUA_{i}-k_{2}US_{i}-\alpha_{1}US_{i}-\delta US_{i}$ (6)

$\frac{dDS_{i}}{dt}=qDA_{i}+k_{2}US_{i}-l_{2}DS_{i}-\alpha_{2}DS_{i}-\delta DS_{i}$ (7)

$\frac{dTS_{i}}{dt}=rTA_{i}+l_{2}DS_{i}+nVS_{i}-mTS_{i}-\alpha_{3}TS_{i}-\delta TS_{i}$ (8)

$\frac{dVS_{i}}{dt}=mTS_{i}-nVS_{i}-sVS_{i}-\alpha_{4}VS_{i}-\delta VS_{i}$ (9)

$\frac{dE_{i}}{dt}=\alpha_{1}US_{i}+\alpha_{2}DS_{i}+\alpha_{3}TS_{i}+\alpha_{4}VS_{i}$ (10)

$N=\sum_{i} S_{i}+\sum_{i} UA_{i}+\sum_{i} DA_{i}+\sum_{i} TA_{i}+\sum_{i} VA_{i}+\sum_{i} US_{i}+\sum_{i} DS_{i}+\sum_{i} TS_{i}+\sum_{i} VS_{i}$ (11)

Healthy people (Susceptible), Undiagnosed (UA and US), Diagnosed not on ART (DA and DS), on ART, not VLS (TA and TS), VLS patients (VA and VS), and HIV-related death (D) represent the compartments in S2 Fig and correspond the system of differential equations (Equation 1-11) in the model formulation. The number of people in each compartment is presented as a function in time in each equation. Disease stages are also taken into account based on the CD4 level as follows: patients with CD4≥200 cells/mm^3^ are included in the UA, DA, TA, and VA compartments, and patients with CD4 <200 cells/mm^3^ are included in the US, DS, TS, VS compartments. Intuitively, each change is calculated by multiplying the number of people in each compartment by the corresponding rate. In all equations, there is index *i* which shows the risk groups [men who have sex with men (MSM), people who inject drugs (PWID), and heterosexuals (HET)].

The movements between the compartments are illustrated with notations as given in the figure. When a healthy individual in the S compartment meets patients from the other compartments, they get infected with HIV and move from the S compartment to the undiagnosed (UA) compartment as they do not know that they are infected. Thus, HIV transmission between susceptible people and people living with HIV is controlled by the force of infection rates (β_1_, β_2_, β_3_) in equations 1 and 2. Individuals get tested and move from the UA to the DA and from the US to the DS compartments with the testing and diagnosis rates of *k_1_* and *k_2_*, respectively. Diagnosed patients can receive treatment with the rates of *l_1_ and l_2_* and move from the DA to the TA and from the DS to the TS compartments, respectively. The movements between TA and VA and between TS and VS are assumed to be reciprocal. *m* shows the VLS rate among patients on ART while dropping out of VLS is included in the model with the rate of *n*. Besides, the progression of the disease is considered in the equations using progression rates (*p,q,r,* and *s*) for each compartment. The population is not constant with birth (σ) and death rates. Death occurs with HIV-related death rates (α_1_, α_2_, α_3_, α_4_) and patients move to the ‘death due to HIV’ compartment; while non-HIV related deaths are shown with δ and applied for all the compartments. The total population size is given in the last equation.

**Initial Population Creation**

The initial population diagnosed for 2005, which was shown in the equations as k3, was taken from the calibration results. With the help of this parameter, we calculated the sum of the number of diagnosed and undiagnosed cases, which was considered the total number of patients living with HIV (PLWH). Further, we needed to distribute these values among risk groups defined as MSM, PWID, and HET in our model. For this, we used the prevalence rates for MSM and PWID in the literature and the rate of transmission ways such as homosexual/bisexual/heterosexual intercourse and injection drug use from the databases of HIV-IST and HIV-Tr cohorts. First, weights were defined for each risk group to find the number of PLWH for each risk group. Prevalence rates were used to find the total number of people in each risk group because population statistics for MSM and PWID are lacking, While calculations were made, we assumed that after excluding the MSM and PWID populations from the total, the remaining part would belong to the HET group.

With the purpose of finding the initial number of people in each compartment, we used the equations below. These equations were performed for 3 risk groups and they were indicated by index i. Rate A and Rate S were the rate of patients with a CD4 level greater than 200 and less than 200 cells/mm^3^, respectively. Since the number of patients categorized by their CD4 level in the data sets in 2005 was not adequate to run the analysis, the initial population was distributed by using 2005 statistics from the Ministry of Health (Tümer, 2014)**.** l1 and l2 were annual treatment rates while o shows the difference between the rate of virally suppressed (VLS) and the rate of dropping out of VLS. In the equations, as the susceptible (S) compartment comprised healthy people, subtracting the total number of living patients from the overall population aged 15-64 gave us the number of people in the S compartment. The number of people in the remaining compartments was found using these parameters in the model. The initial model assumed that compartment E had zero participants. The following calculations such as the annual number of deaths were made based on this assumption.

$$S_{i}={population}_{i}-{living\_patient}_{i}$$

$${UA}_{i}=\frac{1-k_{3}}{k_{3}}*{living\_patient}_{i}*k_{3}* rate\_A$$

$${DA}_{i}=(1-l_{1})*{living\_patient}_{i}*k_{3}* rate\_A$$

$${TA}_{i}=l_{1}*{\left( 1-o \right)*living\_patient}_{i}*k_{3}* rate\_A$$

$${VA}_{i}=l_{1}*{o*living\_patient}_{i}*k_{3}* rate\_A$$

$${US}_{i}=\frac{1-k_{3}}{k_{3}}*{living\_patient}_{i}*k_{3}* rate\_S$$

$${DS}_{i}=(1-l_{2})*{living\_patient}_{i}*k_{3}* rate\_S$$

$${TS}_{i}=l_{2}*{\left( 1-o \right)*living\_patient}_{i}*k_{3}* rate\_S$$

$${VS}_{i}=l_{2}*{o*living\_patient}_{i}*k_{3}* rate\_S$$

**Parameters Estimation**

This section shows the details of calculations for related parameters in the model. To obtain the calculated parameters, we took the weighted average of values found from each data set for the related parameters. Weights were assumed to be the number of patients in that data set.

*Initial Population Parameters*

$$Weights of MSM=\frac{Homosexual/Bisexual Transmission}{Total patients in data}$$

$$Weights of PWID=\frac{Transmission By Injection Drug}{Total patients in data}$$

$$Weights of HET=\frac{Heterosexual Transmission}{Total patients in data}$$

$$People Living with HIV for risk group i=Weight i*Total People Living with HIV$$

$$Diagnosed patients for i=People Living with HIV for i*Diagnosis rate$$

$$Population size for risk group i=\frac{People living with HIV for risk group i}{HIV prevalence for risk group i}$$

*Continuum of Care Parameters*

$$Percentage of Patients on ART=\frac{Number of Patients on ART}{Total patients in data}$$

$$Percentage of Patients on VLS=\frac{Number of Patients on VLS}{Total patients in data}$$

$$Percentage of dropping out of VLS=\frac{Number of Patients dropping out VLS}{Total patients on VLS in data}$$

Patients with a viral load of less than 1000 copies/mL at week 48 of treatment were considered virally suppressed patients in this study. A patient with VLS at week 24 of treatment but not virally suppressed on week 48 was considered to drop out of VLS.

$$HIV mortality rate among specified compartment=\frac{Deaths due to HIV}{Total patients in data}$$

HIV mortality rates were calculated for different stages of the disease such as undiagnosed people, people diagnosed with HIV but not on ART, people on ART, and people with VLS. The main issue here was that we excluded deaths with unknown causes, deaths with suicide, deaths with an accident, etc. in the data sets.

$$Percentage of determined CD4 levels=\frac{Number of Patients with that CD4 level}{/Total patients with known CD4 level}$$

Having percentages of CD4 levels was very useful in cost calculations as costs provided by the literature included costs based on different CD4 levels than those used in our study. As an initial phase of costs calculations, we divided the data sets into 4 CD4 levels (less than 100, between 100 and 200, between 200 and 300, and greater than 300 cells/mm^3^) and determined their percentages.

$$Rate=1-e^{-\frac{1}{time}}$$

As for disease progression rates, we benefited from the exponential distribution formula and converted the time value into a rate.

$$HIV prevalence for PWID=\frac{Number of HIV patients among PWID}{Number of PWIDs tested for HIV}$$

The prevalence rate for PWID was calculated using the Turkey Drug Report results for those receiving treatment for drug use (İçişleri Bakanlığı, Türkiye Uyuşturucu ve Uyuşturucu Bağımlılığı İzleme Merkezi, 2013).

**Model calibration**

At the first stage of the calibration process, the authors decided on the parameters to be included in the analysis. The force of infection, which was a 3x3 matrix, the distribution of the initial population diagnosed for 2005 (range 0.25-0.7), the rate of disease improvements for the three compartments (range 0.02-0.09), and the annual diagnosis rates (range 0.01-0.5) were calibrated in this study. These parameters were calibrated using the targeted number of diagnosed cases by the CD4 level. We created target values by multiplying the total number of diagnosed patients reported by the Ministry of Health with percentages for CD4 levels less (greater) than 200 obtained from the HIV-IST and the HIV-TR data sets. We initiated the first stage of the calibration by running the model 10,000 times between the years 2005 and 2012 and calibrated the parameters based on 2010-2011 targets. Mean squared errors (MSE) were calculated by taking the difference between the diagnosed number of cases in 2010 and 2011 and the target values to reach the targets as close as possible. Then, another 10,000 runs were performed for 2013-2019 with the aim of reaching the 2017-2018 targets. Parameters were selected based on the ranking of results according to the MSE values. Then, the model was run 10,000 times in order to narrow the range of the parameters. Based on the results, the calibrated values of parameters for the force of infection, the distribution of the initial population diagnosed for 2005, the rate of disease improvements for the three compartments, and the annual diagnosis rates were obtained.

Furthermore, the second stage of calibration was applied to estimate the number of HIV-related deaths. Thus, the number of deaths due to HIV, which was reported by the Ministry of Health, was considered a target at this stage. It was performed with 5,000 runs based on the 2017-2018 targets.

$$MSE=\frac{1}{n}\sum_{i=1}^{n} \left( Y_{i}-\hat{Y}_{i} \right)^{2}$$

Ranges for death rates were determined using the number of deaths and their CD4 levels in the data sets. Death rates were calibrated for each compartment the CD4 level is less than 200. Based on the mean squared error calculations, the appropriate result and its parameters were recorded for the next steps of the model runs. All calibration results were given in Table 2. After the calibration process was ended, the model was run by using the calibrated parameters for the prediction period (2020-2040).

**(a) (b)**


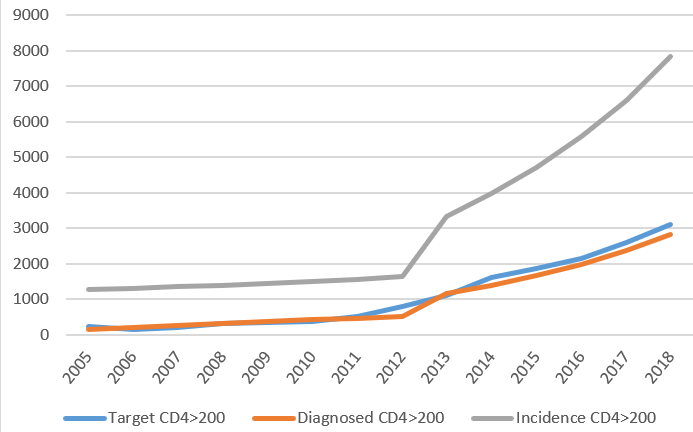

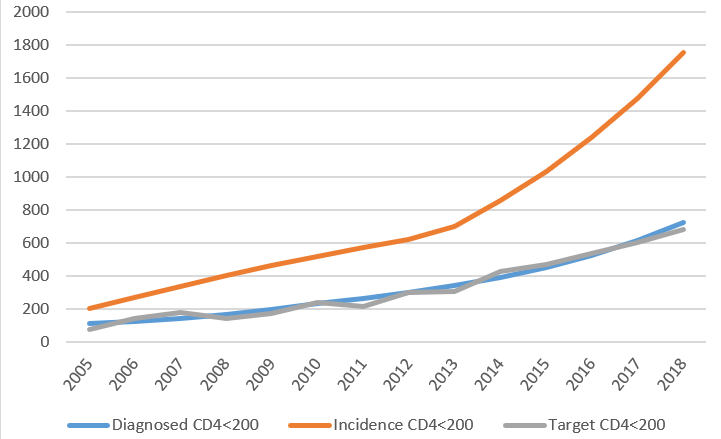


**S3 Fig.** Model results compared to Ministry of Health data, 2005-2018 (a) For CD4≥200 cells/mm^3^ (b) For CD4 <200.

**Sensitivity Analysis:**

One-Way Sensitivity analysis was performed by changing only one parameter and running the model with this new parameter set. To begin, the parameters were selected to be included in the sensitivity analysis and then the decision was to use total incidence during 2020-2040 as an output of the model. Tornado graphs were drawn taking the difference between the output of the base case and the output of the new parameter set one by one. In this way, it became easier to see the effect of the parameter that changed in each parameter set.

**S4 Fig.** Tornado diagram showing bottom 10 important parameters Abbreviations: VLS: Viral load suppression, ART: Antiretroviral treatment.

The elementary effects method (Morris Method) was conducted as a second sensitivity analysis in the study because of the efficient structure of the algorithm. The first step was the definition of the parameters, their ranges, and distributions. We selected 22 parameters, which could be between 0-1, and assumed that they followed a uniform distribution in the range. We focused on the force of infection parameter for each risk group due to the design of the method. After that, the following steps were applied:

- The range was divided into bins of equal sizes. The number of bins was the same as the number of iterations to perform. The number of iterations was chosen as 15 in this analysis.
- We benefited from the Latin Hypercube Sampling (LHS) process. Thus, parameter values were randomly generated from each bin and the iterations were designed by selecting the value of the parameter from each bin without any repetition. These parameter sets can be regarded as the initial values of parameters, or in other words, the base value of iterations, which were used in the next steps.
- Using the base values of parameters for iteration number one, the output was calculated. At this stage, again the output equals the total incidence during 2020-2040.
- The base value of the first parameter was changed by 20% of the range’s width. While the direction of the change was selected randomly, the direction was readjusted to keep the new value of the parameter within the range. The outcome of the new parameter set was found to make elementary effects calculations.
- Then, the iteration steps were performed from the last values of the created parameters. In other words, after changing the first parameter, the second parameter was changed keeping the current values of the other parameters the same. After the second parameter was changed, the output was re-recorded. This process was applied until all the parameters had changed under the first iteration.
- The remaining analyses were implemented based on repeating the same process for all iterations.
- The results of all iterations were used to calculate the elementary effects of each parameter. The difference in the output divided by the rate of change gives the elementary effect of the related parameter. The following formula shows the way of calculating the elementary effects:

${EE}_{i,n}= \frac{(Y_{i,n}- Y_{i,n-1})}{\Delta}$

- In order to interpret the results, two important measures were calculated: the mean elementary effects and the standard deviation of elementary effects. Elementary effects can be positive and negative depending on the direction of change in the parameter. To prevent canceling out of positive values with negative ones, the literature suggests using the absolute elementary effects. The formulations used in this step were as follows:

$${\mu_{n}}^{*}= \frac{\sum_{i=1}^{I} \left| {EE}_{i,n} \right|}{I}$$

$$\sigma_{n}= \sqrt{\frac{\sum_{i=1}^{I} {({EE}_{i,n}-\bar{{EE}_{n}})}^{2}}{I}}$$

- In the Morris method of the elementary effects sensitivity analysis, the final step was presenting the two measures as a graph where the x-axis represents the mean absolute elementary effects (μ*), while the y-axis shows the standard deviation (σ). The results were interpreted according to the graph.

**Sensitivity analysis with discounting factor:**

In the main cost-effectiveness analysis, we used a 3% discount rate as it complies with the recommendations in the literature. Although using the 3% rate is a common approach, applying alternative discount rates is also suggested. Thus, we ran a sensitivity analysis between different options such as no discounting and a 5% discount rate (S5 and S6 Tables).

**S5 Table.** Cost-effectiveness of improving the percentage of diagnosed HIV-positive persons to 50%, 70%, and 90% by 2024 without discounting.


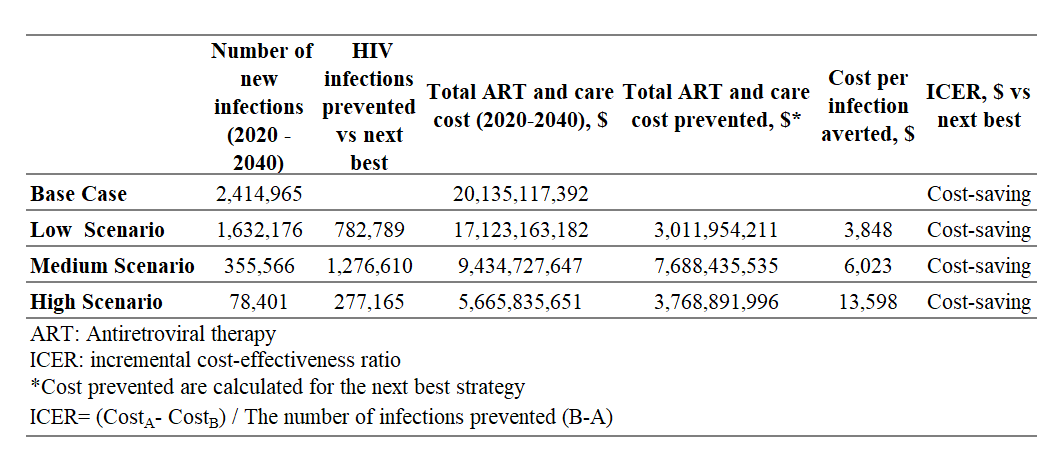


**S6 Table.** Cost-effectiveness of improving the percentage of diagnosed among HIV positive persons to 50%, 70%, and 90% by 2024 with a 5% discount rate


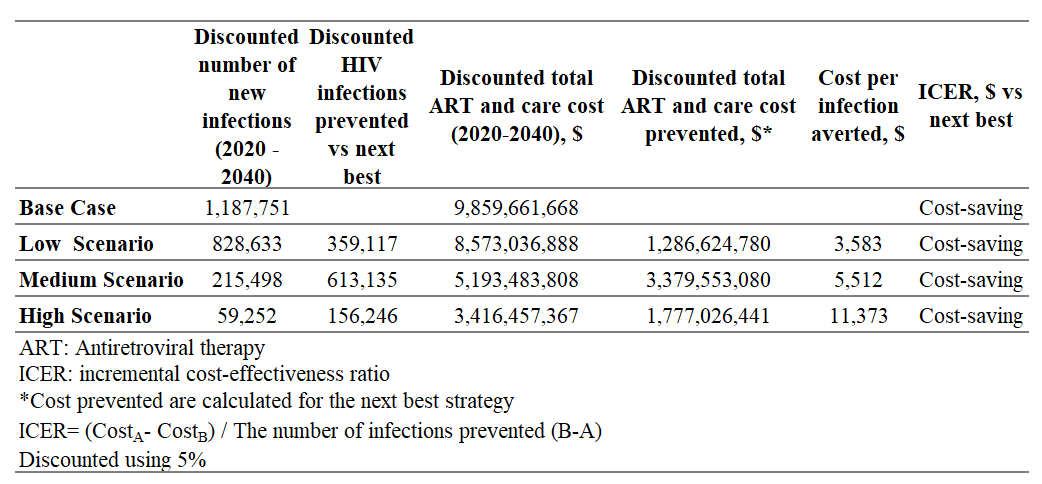


**Model results with indirect costs:**

To provide a larger perspective on cost, i.e. a truncated societal perspective, we added the results with the inclusion of indirect costs below. We calculated ICERs where the costs included productivity loss cost due to HIV and presented these results in the S7 Table.

**S7 Table.** Cost-effectiveness of improving the percentage of diagnosed HIV-positive persons to 50%, 70%, and 90% by 2024 with direct and indirect costs.


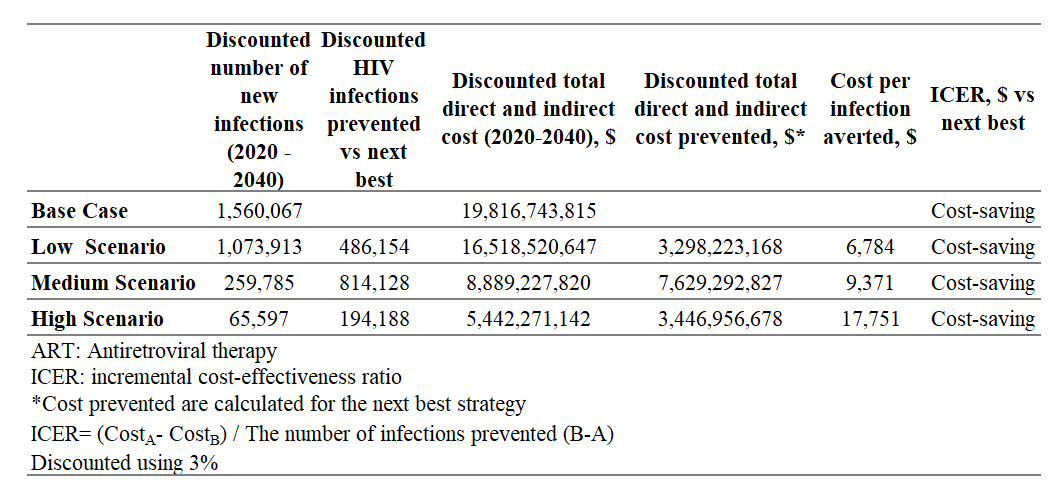

Supplement: S1 File — More detailed information, such as model diagram and explanation, calibration process and cost calculations, was summarized in S1 File. (DOCX) [file pone.0286254.s001.docx]
