# Supplementary material for: Modeling the future of HIV in Turkey: Cost-effectiveness analysis of improving testing and diagnosis
Source: PLoS One. 2023 Jun 30;18(6):e0286254. doi: 10.1371/journal.pone.0286254 (PMC10313051; doi:10.1371/journal.pone.0286254)
Supplement: S2 File — (DOCX) [file pone.0286254.s002.docx]

**CHECKLIST FOR ECONOMIC EVALUATION**

**CHEERS STATEMENT**

**CHEERS checklist—Items to include when reporting economic evaluations of health interventions**

| **Section/item** | **Item No** | **Recommendation** | **Reported on page No/ line No** |
| --- | --- | --- | --- |
| **Title and abstract** | | | |
| Title | 1 | Identify the study as an economic evaluation or use more specific terms such as “cost-effectiveness analysis”, and describe the interventions compared. | Page no. 01, Line no. 02 to 03 |
| Abstract | 2 | Provide a structured summary of objectives, perspective, setting, methods (including study design and inputs), results (including base case and uncertainty analyses), and conclusions. | Page no. 03, Line no. 51 to 77 |
| **Introduction** | | | |
| Background and objectives | 3 | Provide an explicit statement of the broader context for the study. | Page no. 04, Line no. 78 to 108  Page no. 05, Line no. 109 to 117 |
|  |  | Present the study question and its relevance for health policy or practice decisions. | Page no. 05, Line no. 118 to 124 |
| **Methods** | | | |
| Target population and subgroups | 4 | Describe characteristics of the base case population and subgroups analysed, including why they were chosen. | Page no. 05, Line no. 130 to 135 |
| Setting and location | 5 | State relevant aspects of the system(s) in which the decision(s) need(s) to be made. | Page no. 05. Line no. 127 to 130 |
| Study perspective | 6 | Describe the perspective of the study and relate this to the costs being evaluated. | Page no. 09, Line no. 186 to 187 |
| Comparators | 7 | Describe the interventions or strategies being compared and state why they were chosen. | Page no. 10, Line no. 198 to 202 |
| Time horizon | 8 | State the time horizon(s) over which costs and consequences are being evaluated and say why appropriate. | Page no. 10, Line no. 202 to 204 and  Page no. 10 Line no. 219 to 220 |
| Discount rate | 9 | Report the choice of discount rate(s) used for costs and outcomes and say why appropriate. | Page no. 10, Line no. 220-221 |
| Choice of health outcomes | 10 | Describe what outcomes were used as the measure(s) of benefit in the evaluation and their relevance for the type of analysis performed. | Page no. 10, Line no. 205 to 209  Page no. 10, Line no. 217 to 219 |
| Measurement of effectiveness | 11a | *Single study-based estimates:*Describe fully the design features of the single effectiveness study and why the single study was a sufficient source of clinical effectiveness data. | Not applicable |
|  | 11b | *Synthesis-based estimates*: Describe fully the methods used for identification of included studies and synthesis of clinical effectiveness data. | Page no. 07, Line no. 174 to 182  Table 1 |
| Measurement and valuation of preference based outcomes | 12 | If applicable, describe the population and methods used to elicit preferences for outcomes. | Not applicable |
| Estimating resources and costs | 13a | *Single study-based economic evaluation:* Describe approaches used to estimate resource use associated with the alternative interventions. Describe primary or secondary research methods for valuing each resource item in terms of its unit cost. Describe any adjustments made to approximate to opportunity costs. | Not applicable |
|  | 13b | *Model-based economic evaluation:*Describe approaches and data sources used to estimate resource use associated with model health states. Describe primary or secondary research methods for valuing each resource item in terms of its unit cost. Describe any adjustments made to approximate to opportunity costs. | Page no. 06, Line no. 155 to 169 and  Page no. 07, Line no. 170 to 182  Page no. 09, Line no. 185 to 196  Table 1  Supplemental Appendix Table S.1-4 and Page 3-4 |
| Currency, price date, and conversion | 14 | Report the dates of the estimated resource quantities and unit costs. Describe methods for adjusting estimated unit costs to the year of reported costs if necessary. Describe methods for converting costs into a common currency base and the exchange rate. | Page no. 09, Line no. 186 to 193  Supplemental Appendix Table S.2 |
| Choice of model | 15 | Describe and give reasons for the specific type of decision-analytical model used. Providing a figure to show model structure is strongly recommended. | Page no. 05, Line no. 126 to 138  Page no. 06, Line no. 139 to 140  Supplemental Appendix Figure S.2 |
| Assumptions | 16 | Describe all structural or other assumptions underpinning the decision-analytical model. | Page no. 06, Line no. 141 to 154 |
| Analytical methods | 17 | Describe all analytical methods supporting the evaluation. This could include methods for dealing with skewed, missing, or censored data; extrapolation methods; methods for pooling data; approaches to validate or make adjustments (such as half cycle corrections) to a model; and methods for handling population heterogeneity and uncertainty. | Page no. 10 to 11, Line no. 210 to 229  Table 1  Supplemental Appendix Page no. 6 to 10 |
| **Results** | | | |
| Study parameters | 18 | Report the values, ranges, references, and, if used, probability distributions for all parameters. Report reasons or sources for distributions used to represent uncertainty where appropriate. Providing a table to show the input values is strongly recommended. | Page no. 07, Line no. 174 to 182  Table 1 |
| Incremental costs and outcomes | 19 | For each intervention, report mean values for the main categories of estimated costs and outcomes of interest, as well as mean differences between the comparator groups. If applicable, report incremental cost-effectiveness ratios. | Page no. 11 to 12, Line no. 230 to 269  Figure 1  Figure 2  Table 2  Figure 3 |
| Characterising uncertainty | 20a | *Single study-based economic evaluation:* Describe the effects of sampling uncertainty for the estimated incremental cost and incremental effectiveness parameters, together with the impact of methodological assumptions (such as discount rate, study perspective). | Not applicable |
|  | 20b | *Model-based economic evaluation:*Describe the effects on the results of uncertainty for all input parameters, and uncertainty related to the structure of the model and assumptions. | Page no. 12 to 13, Line no. 269 to 287  Figure 4  Figure 5  Supplemental Appendix  Figure S.4 |
| Characterising heterogeneity | 21 | If applicable, report differences in costs, outcomes, or cost-effectiveness that can be explained by variations between subgroups of patients with different baseline characteristics or other observed variability in effects that are not reducible by more information. | Not applicable |
| **Discussion** | | | |
| Study findings, limitations, generalisability, and current knowledge | 22 | Summarise key study findings and describe how they support the conclusions reached. Discuss limitations and the generalisability of the findings and how the findings fit with current knowledge. | Page no. 13 to 16, Line no. 288 to 386 |
| **Other** | | | |
| Source of funding | 23 | Describe how the study was funded and the role of the funder in the identification, design, conduct, and reporting of the analysis. Describe other non-monetary sources of support. | Page 02, Line no. 31 |
| Conflicts of interest | 24 | Describe any potential for conflict of interest of study contributors in accordance with journal policy. In the absence of a journal policy, we recommend authors comply with International Committee of Medical Journal Editors recommendations. | Page 02, Line no. 34 to 36 |

For consistency, the CHEERS statement checklist format is based on the format of the CONSORT statement checklist
