# Supplementary material for: Modeling the future of HIV in Turkey: Cost-effectiveness analysis of improving testing and diagnosis
Source: PLoS One. 2023 Jun 30;18(6):e0286254. doi: 10.1371/journal.pone.0286254 (PMC10313051; doi:10.1371/journal.pone.0286254)
Supplement: Attachment — Submitted filename: Summary comments to authors.docx [file pone.0286254.s004.docx]

**Summary comments to authors**

The effectiveness was established through mathematical modelling of the different scenarios, including the status quo. No randomized controlled trial was done. This therefore may not present strong evidence of effectiveness. A randomized controlled trial would provide a gold standard. In addition, the authors would do well to provide justification of relying on mathematical modelling. The range of costs were presented in detail for all scenarios including the status quo. The costs were also wide enough for the research question. However, it would also have been helpful to have cost from the provider perspective, as the focus for these costs were from the payer’s perspective.

This would have provided enough evidence for real-world decision making. The paper covered the payer’s viewpoint, but did not cover the other relevant viewpoints, such as the community and providers. If these viewpoints were covered, then they should be clear and obvious to the readers. This information is important so as to get as much information as possible to make a strong inference. This may have had possible effects on the ICER. Capital costs and operating costs such as salaries for staff were not included. The study took into account costs of consumables such as test kits for CD4 and viral load and the antiretroviral medicines. It is important that the authors also revisit this particular aspect of the analysis, which may have an effect on the final result.
